# Laparoscopic partial splenectomy for splenic lymphangioma: a case report

**DOI:** 10.1186/s40792-020-00882-1

**Published:** 2020-06-18

**Authors:** Kotaro Kimura, Yo Kurashima, Kimitaka Tanaka, Yoshitsugu Nakanishi, Toshimichi Asano, Yuma Ebihara, Takehiro Noji, Soichi Murakami, Toru Nakamura, Takahiro Tsuchikawa, Keisuke Okamura, Toshiaki Shichinohe, Hiromi Kanno-Okada, Satoshi Hirano

**Affiliations:** 1 Department of Gastroenterological Surgery II, Hokkaido University Faculty School of Medicine, North 15 West 7, Kita-ku, Sapporo, Hokkaido 0608638 Japan; 2 Department of Surgical Pathology, Hokkaido University Hospital, North 15 West 7, Kita-ku, Sapporo, Hokkaido 0608638 Japan

**Keywords:** Laparoscopic partial splenectomy, Splenic benign tumor, Splenic lymphangioma

## Abstract

**Background:**

Lymphangioma is a benign malformation of the lymphatic system and is often found in the neck and axilla, the orbit, the mediastinum, etc. However, isolated splenic lymphangioma is a rare disease in young women, and its treatment is controversial. We report a case of laparoscopic partial splenectomy for isolated splenic lymphangioma in a young woman.

**Case presentation:**

An 18-year-old woman with mild epigastralgia was admitted to a nearby hospital. Abdominal ultrasound detected a 6-cm mass confined to the upper pole of the spleen; thereafter, she was referred to our department for surgical treatment. Although a benign tumor, we decided to resect it because of her symptoms. To preserve part of the normal spleen, laparoscopic partial splenectomy was performed with a co-axial approach using four ports and a liver retractor in the lithotomy position. After dissection around the spleen hilum, we identified that the tumor was being fed from the splenic vessels of the upper pole and severed the branch. Postoperatively, the patient showed no complications and was discharged on postoperative day 8 without symptoms. Pathological examination revealed splenic lymphangioma, which is rare in young women. No recurrence was seen 1 year after surgery, and a computed tomography scan showed no problems with the remaining spleen.

**Conclusions:**

In our experience of laparoscopic partial splenectomy for a young woman with an isolated splenic lymphangioma, we determined that laparoscopic partial splenectomy is a safe, effective, and valuable option for the treatment of benign splenic tumors.

## Background

Lymphangioma is a benign malformation of the lymphatic system and is often found in the neck and axilla, the orbit, the mediastinum, the adrenal gland, the kidney, the bone, the omentum, and the gastrointestinal tract [1]. However, isolated splenic lymphangioma is a rare disease in young women, and its treatment is controversial because it is important to preserve the function of the spleen in younger patients. We report a case of laparoscopic partial splenectomy for isolated splenic lymphangioma in a young woman.

## Case presentation

**Fig. 1:**
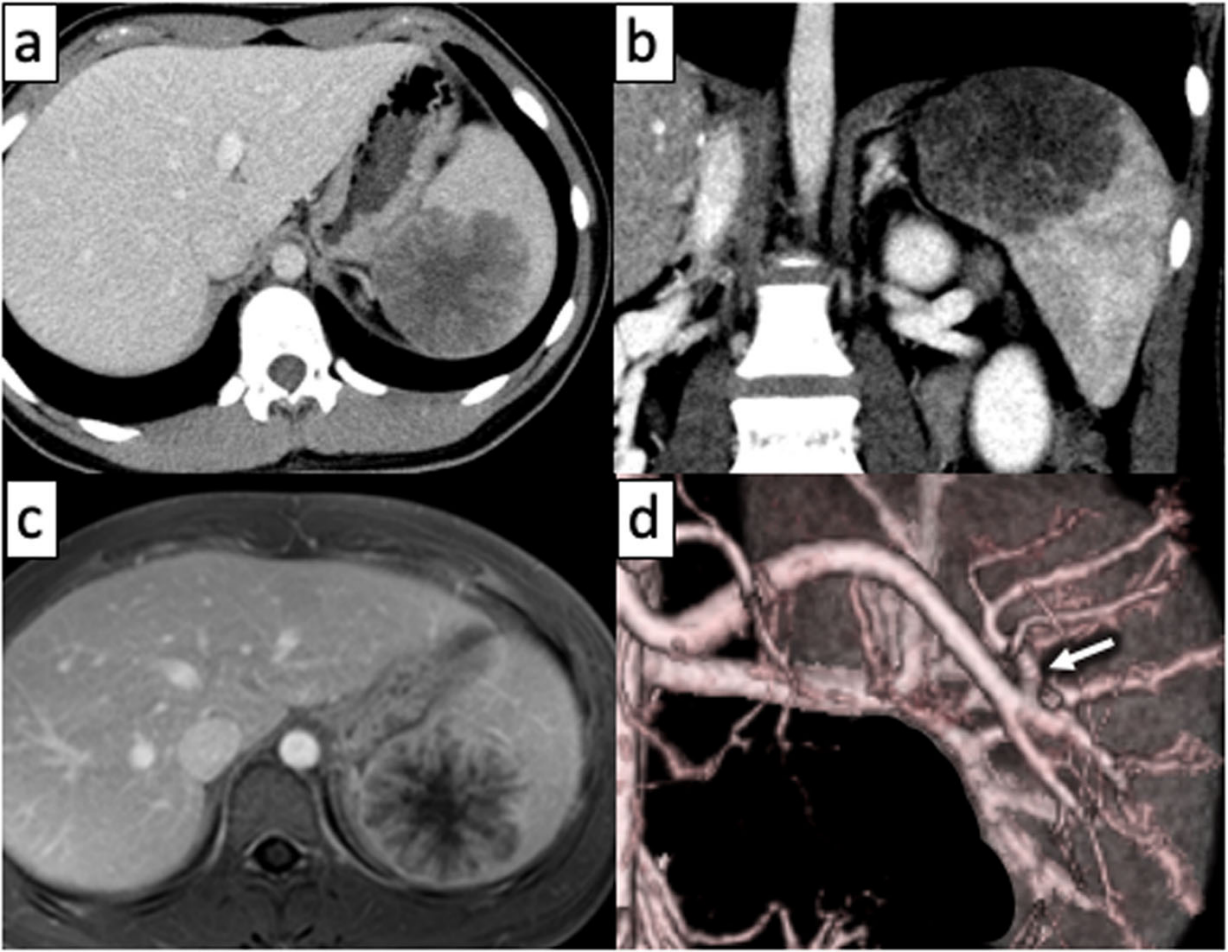
**a**, **b** A CT scan of the abdomen showing a hypodense lesion in the upper pole of the spleen. **c** MRI shows a mass contrasted from edge to center, the so-called spoke-wheel pattern. **d** Angiography showing the upper pole branch of the splenic artery (arrow)

**Fig. 2:**
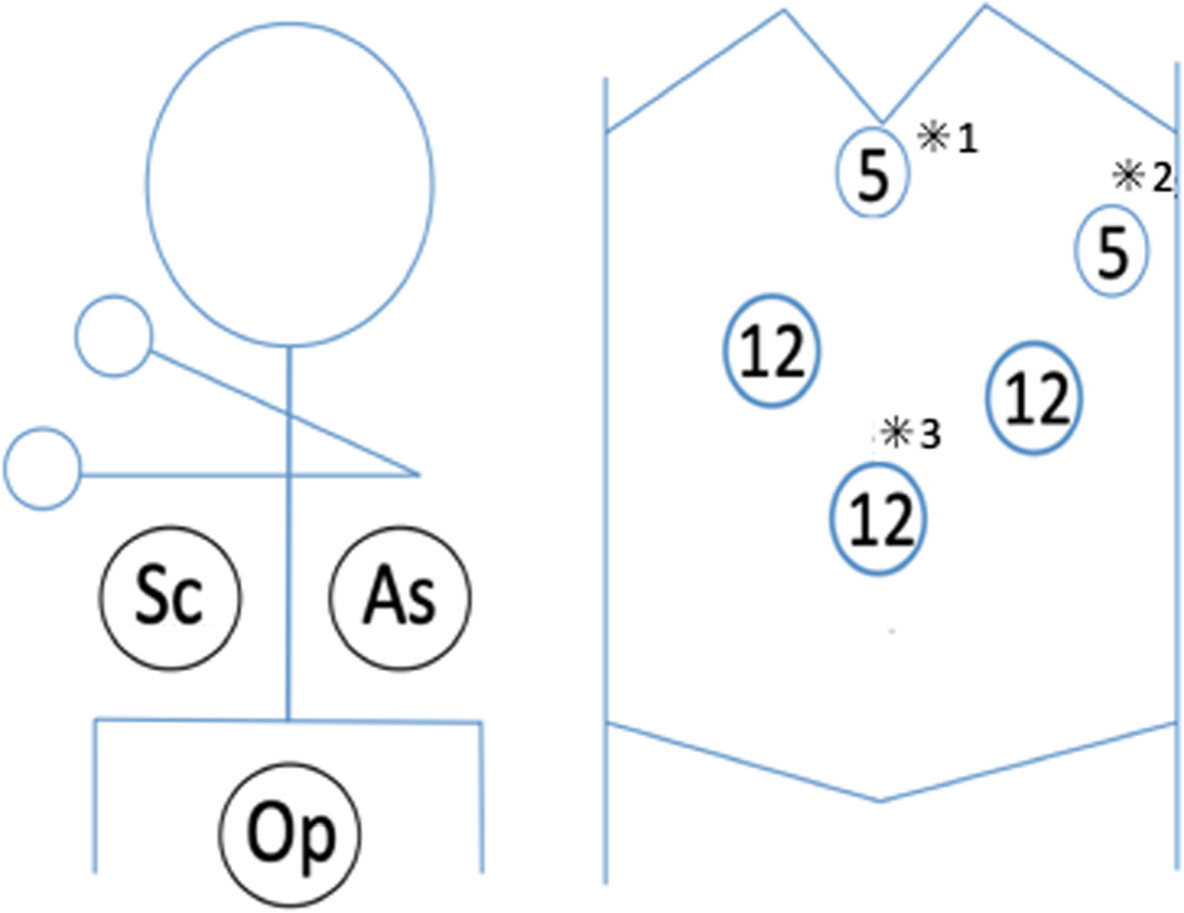
The patient is under general anesthesia in the lithotomy position and undergoes a laparoscopic surgery with a co-axial approach using four ports and a liver retractor (*1). The left hypochondrium port is for the assistant surgeon (*2), and the umbilicus port is for laparoscopy (*3). Op, operator; As, assistant surgeon; Sc, scopist

**Fig. 3:**
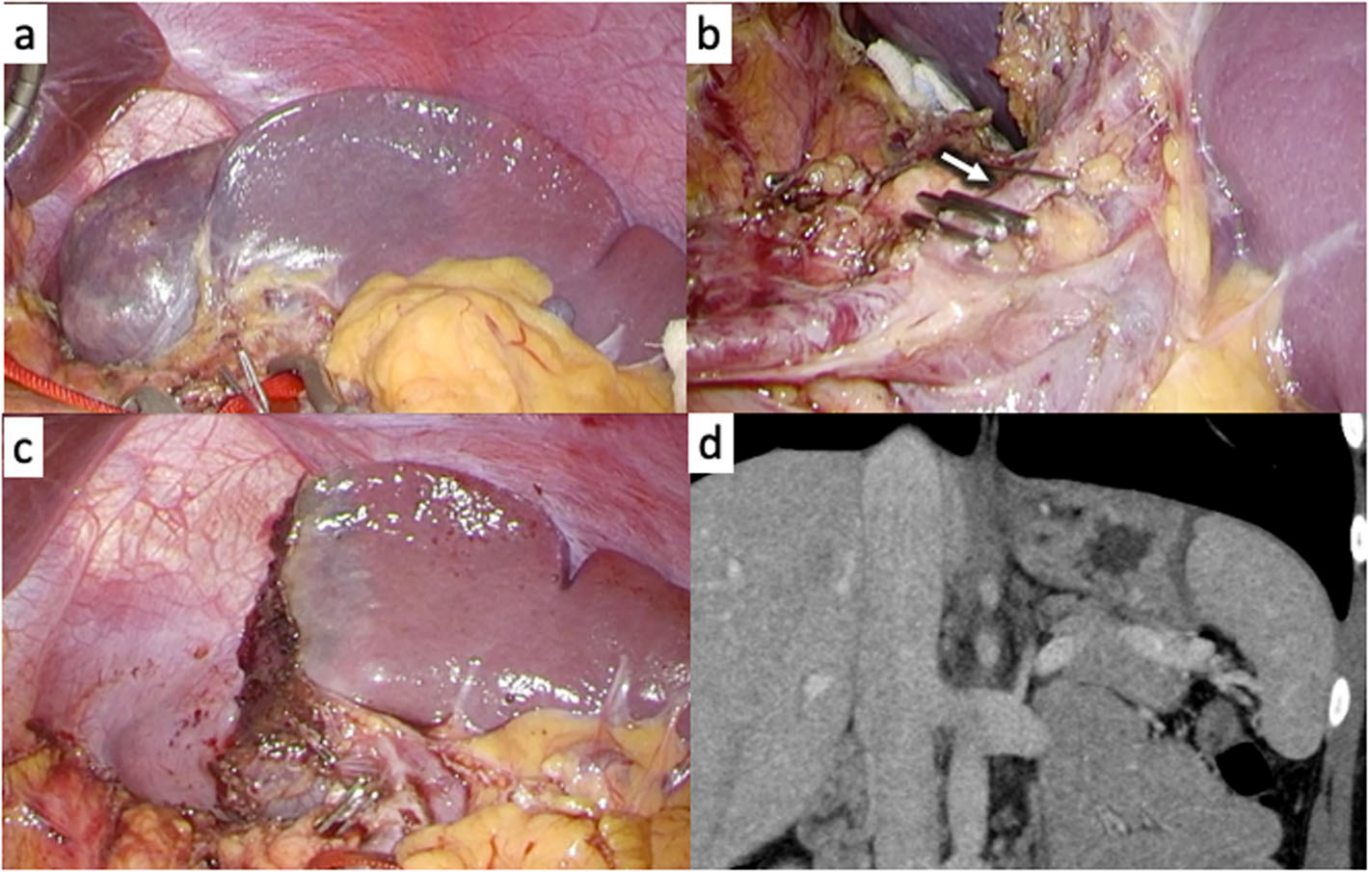
The demarcation line appears after the upper pole branch of the splenic vessels is clamped (**a**). The upper pole branch of the splenic vessels is divided (**b**); the arrow shows the upper pole branch of the splenic vessels. The spleen is dissected approximately 1 cm from the ischemic side of the demarcation line with less bleeding (**c**). Computed tomography a year after surgery shows a spleen with preserved blood flow (**d**)

**Fig. 4:**
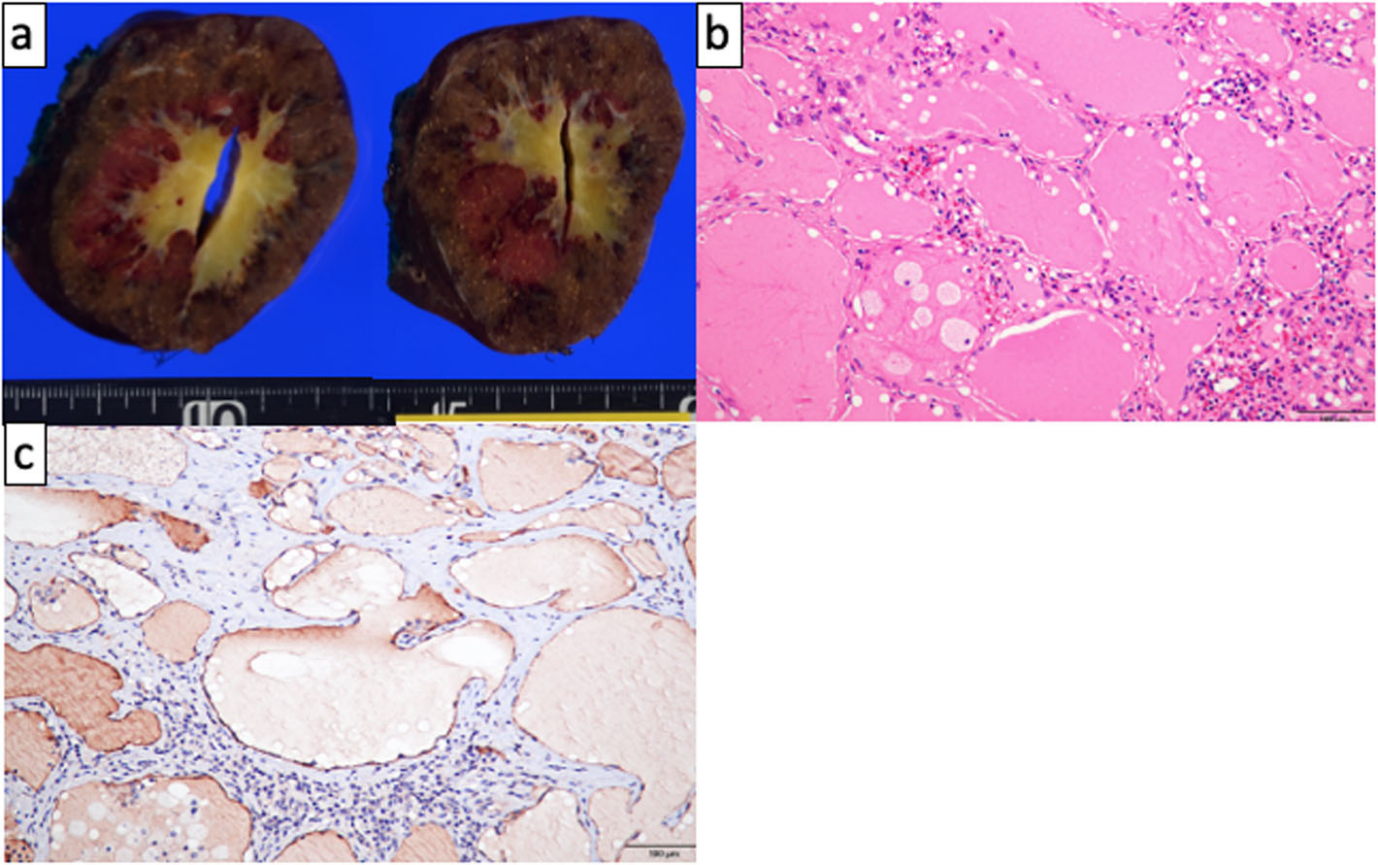
The specimen shows a well-defined mass of 60 × 48 mm (**a**). Histologically, vascular-like structures are growing; in the lumen, there is storage of lightly acidic serous substances and histiocytic cells (**b**). Immunohistochemical staining shows a D2-40 weak positive result (**c**)

The patient was an 18-year-old woman with mild epigastralgia who was admitted to a nearby hospital. An abdominal CT scan showed a 60-mm mass confined to the upper pole of the spleen; therefore, she was referred to our department for surgical treatment (Fig. 1a, b). The tumor showed higher intensity on T1-weighted MRI and lower intensity on T2 imaging than the normal spleen. In dynamic MRI, the contrast was gradually enhanced from the edge toward the inside, with a spoke-wheel pattern, without malignant findings (Fig. 1c). From such findings, the tumor was suspected to be splenic lymphangioma. It was a benign tumor, but we decided to resect it because of her symptoms. We performed laparoscopic partial splenectomy on the patient with the plan to divide the upper pole branch of the splenic artery and remove only the upper spleen (Fig. 1d). The patient underwent surgery under general anesthesia in the lithotomy position by a co-axial approach using four ports and a liver retractor (Fig. 2). After peeling the tissue around the pancreatic tail and spleen hilum, the upper pole branch of the splenic vessels was clamped (Fig. 3a). The ischemic side of the tumor was confirmed, and the upper pole branch of the splenic vessels was divided as planned (Fig. 3b). Using a bipolar electrocoagulation hemostasis device and a vessel sealing system, the spleen was dissected at the ischemic parenchyma approximately 1 cm from the demarcation line. Because the ischemic-side spleen was dissected, there was less bleeding (Fig. 3c). The excised spleen was retrieved from the umbilical wound and extended several centimeters using a retrieval bag without crushing the specimen. The operation time was 217 min, and bleeding was minimal. The patient was discharged after 8 days without complications. The patient’s symptoms disappeared with no recurrence at 1 year postoperatively. Blood tests and CT scans confirmed no problems with the remaining spleen (Fig. 3d). The specimen showed a well-defined mass of 60 × 48 mm (Fig. 4a). Histologically, vascular-like structures were growing, and in the lumen, lightly acidic serous substances and histiocytic cells were stored (Fig. 4b). Immunohistochemical staining yielded D2-40 weakly positive (Fig. 4c), CD31-positive, CD34-negative, and CD8-negative results (data not shown); hence, a cavernous type lymphangioma was diagnosed. Ethics committee approval was unnecessary for our case report, and the patient gave informed consent; anonymity was preserved.

## Discussion

Lymphangioma is a congenital benign disease often found in patients aged up to 2 years. But it can also be seen in young women, with 75% occurring in the neck, 20% in the axilla, and rarely in the abdominal organs [1, 2]. There are few reports of isolated splenic lymphangioma (SL) in young people. The reported cases of isolated SL are summarized in Table 1 [3–15]. There are 15 articles including our case, and 19 patients have been reported. Of those patients, there was only one teenager, other than our case [9].

**Table 1:** Case reports on isolated splenic lymphangioma

Reference	Public year	Age	Gender	Tumor size (mm)	Operation	Pathological diagnosis
[3]	2001	69	F	Multiple	LTS	Lymphangioma
[4]	2007	46	F	40	OTS	Lymphangioma
[5]	2007	26	F	30	LTS	Lymphangioma
		30	F	45	LTS	Lymphangioma
		40	F	60	LTS + LC	Lymphangioma
[6]	2012	59	F	Multiple	LTS	Lymphangioma
[7]	2012	67	M	60	Not done	Lymphangioma
[8]	2013	30	F	N/A	OTS	Lymphangioma
[9]	2014	12	M	157	LPS	Hemolymphangioma
[10]	2014	22	M	42	LPS	Lymphangioma
		56	F	40	LPS	Lymphangioma
		47	F	44	LPS	Lymphangioma
[11]	2015	51	F	15	LPS	Lymphangioma
[2]	2015	41	F	Multiple	LTS	Lymphangioma
[12]	2016	64	F	118	OTS	Lymphangioma
[13]	2017	34	F	30	OTS	Lymphangioma
[14]	2018	40	F	Multiple	OTS	Lymphangioma
[15]	2019	63	M	Multiple	OTS	Lymphangioma
Our case		18	F	60	LPS	Lymphangioma

*OTS* open total splenectomy, *LTS* laparoscopic total splenectomy, *LPS* laparoscopic partial splenectomy, *LC* laparoscopic cholecystectomy

Other organs are often reported to be involved in the alleged lymphangiomatosis syndrome, which can occur in patients with Klippel-Trenaunay syndrome [1, 16, 17]. Therefore, it is recommended to examine the other organs of patients with SL [16]. In this case, CT scan and MRI showed no lesions except in the spleen; hence, it was diagnosed as an isolated SL. Diagnostic ultrasonography showed hypoechoic or anechoic cystic lesions, often accompanied by internal septations and calcification [1, 16]. On CT scans, the lymphangioma appears as a low-density lesion with a thin subcapsular wall. However, if the tumor is large, cystic lesions may have been located within the parenchyma [16, 17]. The presence of curvilinear peripheral mural calcifications strongly suggests the diagnosis of cystic lymphangioma [16]. With T1-weighted MRI, cystic lesions often appeared as a hypointense lesion, but T2-weighted images showed multilocalized, hyperintense areas corresponding to dilated lymphatic spaces [16]. Furthermore, as in this case, a dynamic study described the cavernous type of lymphangioma as having a typical spoke-wheel pattern that is imaged from the margin to the center. Asymptomatic SLs are usually detected by imaging. However, large tumors may cause abdominal pain or abdominal bloating due to compression by the tumor [2, 16, 17]. Additional complications such as bleeding, coagulopathy, hypersplenic function, or portal hypertension may occur from an extremely large tumor [1, 2, 16]. Follow-up is typical for benign splenic tumors, including SL. However, if there are symptoms or a tendency toward tumor growth, surgery is necessary [1, 16, 17].

Although total splenectomy (TS) has been traditionally performed for benign splenic tumors, incidents of serious complications have been reported. In particular, overwhelming post-splenectomy sepsis (OPSS) is the most severe and specific complication after TS, occurring in up to 5.7% of patients after the procedure and has a mortality rate approximately 600 times greater than that of the general population [18, 19]. Therefore, partial splenectomy (PS) should be an alternative to preserve the function of the spleen. Research reports using rats showed that at least 25% of the normal spleen should be preserved to maintain function [20, 21]. We believe that the younger the patient, the more valuable it is to preserve the normal spleen.

In recent years, with the popularization of laparoscopic surgery, there are reports on laparoscopic PS (LPS) for benign splenic tumors. A systematic review of 187 LPS cases [22] showed that LPS is a safer and less invasive technique than open TS, laparoscopic TS, and open PS in terms of bleeding volume, complication rate, laparotomy conversion rate, and postoperative recurrence rate. As shown in Table 1, there are 5 reports of LPS for SL in addition to our case, and there are no recurrences in the reports. Therefore, LPS could be considered as an appropriate alternative for SL treatment.

To preserve the function of the spleen, we chose LPS for benign tumors confined to the upper side of the spleen this time. If the tumor is in the splenic hilum, it may be necessary to perform TS because it is challenging to preserve blood flow in the normal spleen. We clamped the upper pole branch of the splenic vessels once and confirmed that the blood flow in the normal spleen was maintained and then cut off the upper pole branch. According to a previous report, it is crucial to dissect the ischemic parenchyma 1 cm away from the demarcation line to reduce bleeding [23]. We used a vessel sealing system to dissect the parenchyma, and a bipolar electrocoagulation hemostasis device to stop the slight bleeding that resulted. We planned a temporal clamp of the main trunk of the splenic vessel in case of massive bleeding, but this was not necessary.

In our experience with LPS in a young woman with an isolated SL, we determined that LPS is a safe, effective, and valuable option for the treatment of benign splenic tumors.

## Conclusion

We report surgical treatment for a rare case of isolated splenic lymphangioma in a young woman. We thought that LPS is a feasible option for the treatment of benign splenic tumors because it is important to preserve the function of the spleen in younger patients.

## Data Availability

Data sharing is not applicable to this article as no datasets were generated or analyzed during the current study.

## Abbreviations

SL: Splenic lymphangioma
TS: Total splenectomy
OPSS: Overwhelming post-splenectomy sepsis
PS: Partial splenectomy
LPS: Laparoscopic partial splenectomy
OTS: Open total splenectomy
LTS: Laparoscopic total splenectomy
LC: Laparoscopic cholecystectomy
